# Bicuspid Aortic Valve Stenosis: From Pathophysiological Mechanism, Imaging Diagnosis, to Clinical Treatment Methods

**DOI:** 10.3389/fcvm.2021.798949

**Published:** 2022-02-08

**Authors:** Nils Perrin, Réda Ibrahim, Nicolas Dürrleman, Arsène Basmadjian, Lionel Leroux, Philippe Demers, Thomas Modine, Walid Ben Ali

**Affiliations:** ^1^Structural Heart Intervention Program, Montreal Heart Institute, Montreal, QC, Canada; ^2^Cardiology Division, Geneva University Hospitals, Geneva, Switzerland; ^3^Service Médico-Chirurgical, Valvulopathies-Chirurgie Cardiaque-Cardiologie Interventionelle Structurelle, Hôpital Cardiologique de Haut Lévèque, CHU Bordeaux, Bordeaux, France

**Keywords:** bicuspid aortic valve stenosis bicuspid aortic valve, nomenclature, transcatheter aortic valve implantation, sizing approaches, review

## Abstract

Bicuspid aortic valve (BAV) is the most frequent congenital anomaly and has a natural evolution toward aortic regurgitation or stenosis due to the asymmetrical valve function associated with an evolutive ascending aortopathy. Several BAV classifications exist describing the presence and number of raphe, amount and location of calcium, and the symmetry of the functional cusps. The impact of BAV morphology on transcatheter aortic valve implantation (TAVI) outcomes still remains little investigated. Pivotal randomized trials comparing TAVI with surgery have excluded BAV until yet. However, data from registries and observational studies including highly selected patients have shown promising results of TAVI in BAV. With this review, we aimed at describing anatomical and pathophysiological characteristics of BAV, discussing the main aspects to assess diagnostic imaging modalities, and giving an overview of TAVI outcomes and technical considerations specific to BAV morphology.

## Introduction

Transcatheter aortic valve implantation (TAVI) has become the standard of care for patients with symptomatic severe aortic stenosis at intermediate and high surgical risk, especially if suitable from a transfemoral approach, and is considered as a valuable option for patients at low surgical risk (1–6). However, in pivotal randomized trials comparing TAVI with surgery, bicuspid aortic valve stenosis (BAV), either congenital or acquired, has been excluded until yet. BAV is the most frequent congenital anomaly and is found in up to 2.25% of the general population. Its natural evolution toward aortic regurgitation and/or stenosis is mainly due to the asymmetrical valve function associated with an evolutive ascending aortopathy. Moreover, BAV was described in >20% of high-risk elderly patients undergoing surgical aortic valve replacement for aortic stenosis (7). This category of patients would largely be considered for TAVI nowadays. In an analysis from the Society of Thoracic Surgeons (STS)/American College of Cardiology (ACC) Transcatheter Valve Therapy (TVT) registry regarding transcatheter heart valve off-label use, Hira et al. reported that about 2% of patients treated for BAV (8). A higher prevalence of BAV was demonstrated in the Chinese TAVR registries (up to 5.8%) (9). In addition to a possible impact of ethnicity difference in BAV prevalence, the younger and lower risk population included in the Chinese BAV studies may lead to interpretation bias. In the worldwide current trend toward younger patients treated by TAVI, transcatheter heart valve operators will face an increasing number of patients with BAV.

With this review, we aimed at describing anatomical and pathophysiological characteristics of BAV, discussing the main aspects to assess with diagnostic imaging modalities, and giving an overview of TAVI outcomes and technical considerations specific to BAV morphology.

## Nomenclature

**Figure 1:**
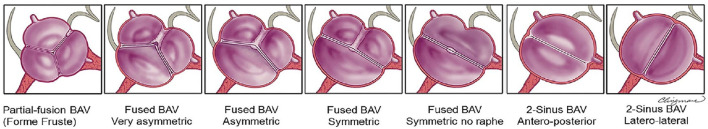
The 2021 international consensus statement on nomenclature and classification of BAV (13).

The BAV is defined by the presence of 2 functional commissures with <3 zones of parallel apposition between them (10). The presence and orientation of the commissural fusion and raphe are highly variable among the population. Fused commissures can be either congenital or acquired through the development of a rheumatological valvular disease or progression of age-related atherosclerosis. In theory, all degrees and combinations of fused cusps can be possible. Most BAV classifications reported in the literature were derived from the surgical analysis yet. Fused commissures most often involve the right and left coronary cusps (80% of the cases), followed by the right and non-coronary cusps and, rarely, the left and non-coronary cusps (10). Sievers is the most widely known and used classification of BAV describing the number and orientation of the raphe based on surgical models (10). Briefly, type 0 has no raphe with 2 normal functioning symmetrical cusps. Type 1 presents one raphe connecting two underdeveloped cusps. Finally, type 2 has two raphes with two underdeveloped cusps and commissures, and one fully developed cusp and commissure. The 2014 International BAV Consortium (BAVCon) adopted a similar but simplified classification system according to the 2 fused cusps. All 3 types (type 1: right-left cusp fusion; type 2: right-non fusion; and type 3: left-non fusion) may or may not have a raphe (11). De Kerchove et al. suggested a classification system assessing the surgical repairability of the BAV, such as commissural orientation (varying from symmetrical to very asymmetrical cusp angles), length of fusion, and non-functional commissure height (12). Very recently, a new international consensus statement on the nomenclature of BAV has been developed with a simple and comprehensive classification system based on imaging modalities (echocardiographic, CT, and MRI) and anatomical surgical pathology (Figure 1) (13). The authors described 3 types of BAV: the fused (similar to Sievers type 1), the 2-sinus (latero-lateral and antero-posterior phenotypes), and the partial-fusion types. The fused-type is thereafter subclassified according to the symmetry of the functional cusps and commissure angle of the non-fused cusp. The present descriptive classification derives from a multidisciplinary consortium and aims at better identifying anatomical features of BAV that best predict the surgical valve replacement or repair success and TAVI outcomes.

**Figure 2:**
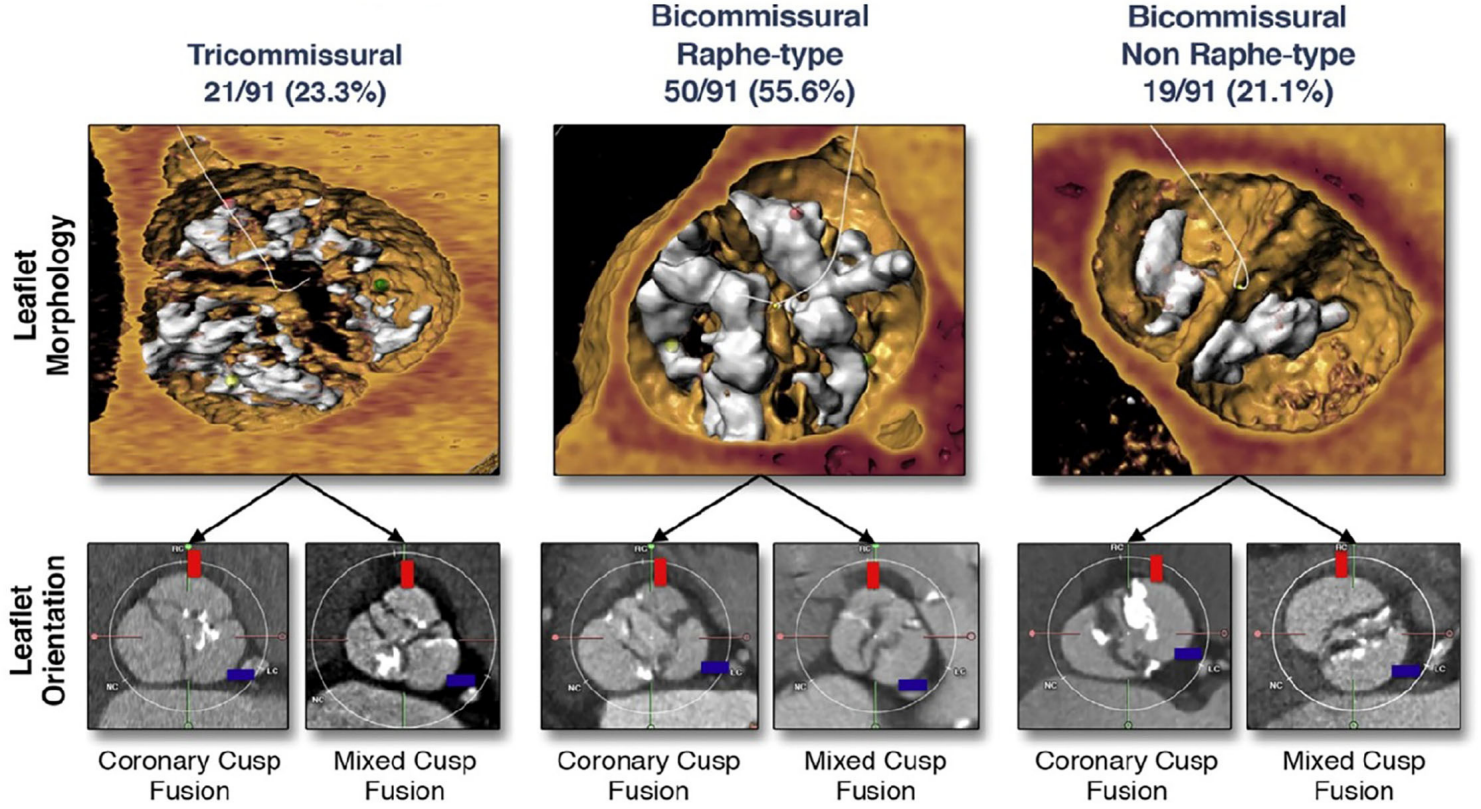
Bicuspid aortic valve classification in the TAVI era (14).

Jilaihawi et al. adapted the traditional Sievers classification to better address the transcatheter heart valve interaction with the aortic root (14). BAV morphologies were defined as bicommissural non-raphe (equivalent to Sievers type 0), bicommissural raphe (equivalent to Sievers type 1), and tricommissural (sharing characteristics between Sievers type 1 and tricuspid valves) types (Figure 2). In an early exploratory study, 30-day mortality, cerebrovascular events, and new pacemaker implantation across the BAV morphologies were similar (14). Interestingly, the intercommissural distance (for bicommissural bicuspids) was associated to ≥moderate paravalvular leak, with respect to the limited power of the study (*n* = 130).

In contrast with the STS Surgical Database Form who started to collect specific anatomical characteristics of BAV in 2017, the large STS/ACC TVT registry does not provide information on BAV sub-type classification. The impact of BAV morphology on TAVI outcomes still remains little investigated yet. In an international multicenter BAV TAVI registry, BAV were classified according to a modified Sievers nomenclature differentiating a calcified raphe to a non-calcified raphe type 1 morphology. Death at 1 year increased significantly between type 0 (no raphe), type 1 with a non-calcified raphe, and type 1 with a calcified raphe (2.4, 4.8, and 9.5%, *p* = 0.006 between the groups, respectively). Moreover, patients with both calcified raphe and excess leaflet calcifications presented significantly higher 2-year mortality and ≥ moderate paravalvular regurgitation in comparison with patients with one or none of these characteristics (15).

## Pathophysiological Characteristics

In comparison with tricuspid valves, BAV has different localization and excess calcification of the aortic valve (16). Asymmetrical BAV leaflet motion and a higher leaflet coaptation point increase the shear stress through the valve leading to a calcification process starting already at a young age. As another consequence of the shear stress, patients with BAV develop progressive aortic root and ascending aorta dilatation. Larger annular and sinus of Valsalva dimensions have been reported among the patient undergoing TAVI for BAV vs. tricuspid valves, respectively (annulus mean area-derived diameter 26.3 ± 3.0 vs. 23.2 ± 1.9 mm, *p* < 0.01 and sinus of Valsalva 930.0 vs. 866.6 mm^2^, *p* = 0.005) (17). Recent MRI blood flow analysis was able to confirm the increased aortic wall shear stress, namely, induced by eccentric jets (11). A small cohort study reported an increased aortic growth associated with the degree of the aortic jet angle (18). Interestingly, other blood flow imaging analysis has suggested a different degree of flow abnormality severity according to the BAV type, thus it remains preliminary investigations (19).

Concomitant congenital anomalies of coronary origin are more frequent with the congenital BAV in comparison with tricuspid aortic valves (7 vs. 3%, *p* = 0.001), affecting mainly anomalous origin of the right coronary artery (20). Whereas, the similar prevalence of anomalous origin of the left main has been observed between BAV and tricuspid valves, the absence of the left main with separate left anterior descending and circumflex artery ostia has been more frequently reported in BAV than tricuspid valves (21). Moreover, from a TAVI perspective, a higher distance from the aortic annulus to coronary ostia has been reported in BAV (22). As discussed later in this review, the origin and height of coronary ostia will be a specific parameter to assess the pre-procedural multislice CT (MSCT).

## Imaging

### Echocardiography

Transthoracic (TTE) and transesophageal echocardiography (TEE) remain the first-line imaging for BAV diagnosis and commissural morphology classification. However, inpatient candidates for TAVI, the important calcification burden of aortic root may limit acoustic windows and participation in misclassification (23). Echocardiography has the best accuracy for aortic valve function analysis. Quantification of BAV aortic stenosis severity is similar to the tricuspid valve and should follow the latest guidelines for valvular heart disease of the European Society of Cardiology (ESC) (24). However, in BAV, maximal velocity flows are most of the time measured at the right parasternal window due to eccentricity of the aortic jet (25). In cases of very eccentric jets, misalignment of the beam leads to maximal velocity underestimation. On the other hand, aortic valve regurgitation severity is more difficult to assess since laminar flow may be falsely assumed at the sinotubular junction leading to inaccurate regurgitation volume calculations. Integration of several parameters, such as aortic holodiastolic retrograde flow velocity, may help to address these limitations. Since BAV is frequently associated with the ascending aorta dilatation, echocardiography often offers favorable visualization of the initial part of the proximal part of the ascending aorta and is thus preferentially used in the clinical practice for patient follow-up.

### Multislice CT

In the current TAVI era, MSCT has an integral part in procedural planning investigations. MSCT has the best accuracy for BAV morphological analysis (26). Detailed analysis of amount and location of aortic root calcification as well as precise aortic and surrounding structures measurements play a pivotal role for prosthesis choice. In comparison with tricuspid valves, BAV has a larger annulus and sinus of Valsalva diameters. In addition, BAV has less elliptical aortic annulus with more eccentric calcifications (27).

### Prosthesis Sizing

Prosthesis sizing is mainly dependent on annular diameter measurement in tricuspid valves. A certain degree of prosthesis oversizing (5–20 and 12–25% for balloon- and self-expandable devices, respectively) is recommended to limit the paravalvular leak and prosthesis embolization (28, 29). Calcified and fibrotic leaflets as well as commissural fusion with or without raphe modify the aortic root anatomy and increase the challenge of valve sizing in BAV. Interaction and interference of the prosthesis with the aortic root can occur from the level of the left ventricular outflow tract to above the sinotubular junction according to the prosthesis design. Balloon sizing with waste measurements and sequential aortography has been suggested by some operators for valve sizing in BAV but has never been meticulously investigated by studies (30). The behavior of calcified leaflets and raphe with respect to the surrounding structures (such as coronary ostia) may also be appreciated during balloon inflation.

Initial evidence from post-TAVI MSCT studies has shown that the maximal stent frame interaction with aortic root in BAV anatomies occurred rather at the supra-annular than annular level, typically between 4 and 8 mm above the annulus (31, 32). Perimeters and area at the supra-annular level will have to be circumscribed by taking into account the border of the leaflets and commissural fusions. Unlike tricuspid valves where the virtual basal ring is easily defined by 3 anatomic distinct hinge points at the nadir part of the cusps, defining the virtual basal ring in BAV is challenging and may lead to inaccurate prosthesis sizing.

Prosthesis sizing according to the level of estimated prosthesis anchoring at a supra-annular plane in raphe-type BAV has been recently suggested by a multicenter MSCT study (33). The so-called level of implantation at the raphe (LIRA) plane is identified where the plane cuts the raphe at the level of its maximum protrusion. The perimeter around the internal border of the leaflet is then traced excluding fused commissures or heavy calcifications. The smallest perimeter between the LIRA plane and the virtual basal ring is then chosen for prosthesis sizing (33). The Calcium Algorithm Sizing for bicusPid Evaluation with Raphe (CASPER) algorithm adapted the perimeter/area derived annulus diameter according to 3 main characteristics: raphe length/annulus diameter ratio, calcium score, and prevalence of calcium distribution on raphe site (34). According to the algorithm, operators detracted 0–2 mm from the area/perimeter derived mean annular diameter for valve sizing. In a validation cohort (*n* = 21), Petronio et al. reported 100% VARC-2 defined procedural success (34).

Even though prosthesis maximal constraint seems to occur at a supra-annular level in imaging studies, the Bicuspid Aortic Valve Anatomy and Relationship with Devices (BAVARD) retrospective registry reported a tapered aortic root configuration (intercommissural distance < annular diameter) in only 13.8% of the BAV raising the question whether supra-annular or annular measurements should be best used for prosthesis sizing (22). Importantly, in this registry, the intercommissural distance was systematically measured 4 mm above the annulus for standardization purposes, leading to a possible higher proportion of tapered configuration according to the level of prosthesis maximal constraints. Tubular (intercommissural distance = annular diameter) and flared (intercommissural distance > annular diameter) configuration accounted for 33.7 and 52.5% of the BAV. According to the BAVARD algorithm, size of the prosthesis should best be chosen according to the smallest measure between the annulus diameter (tubular or flared configuration) or the intercommissural distance (tapered configuration) (22).

**Figure 3:**
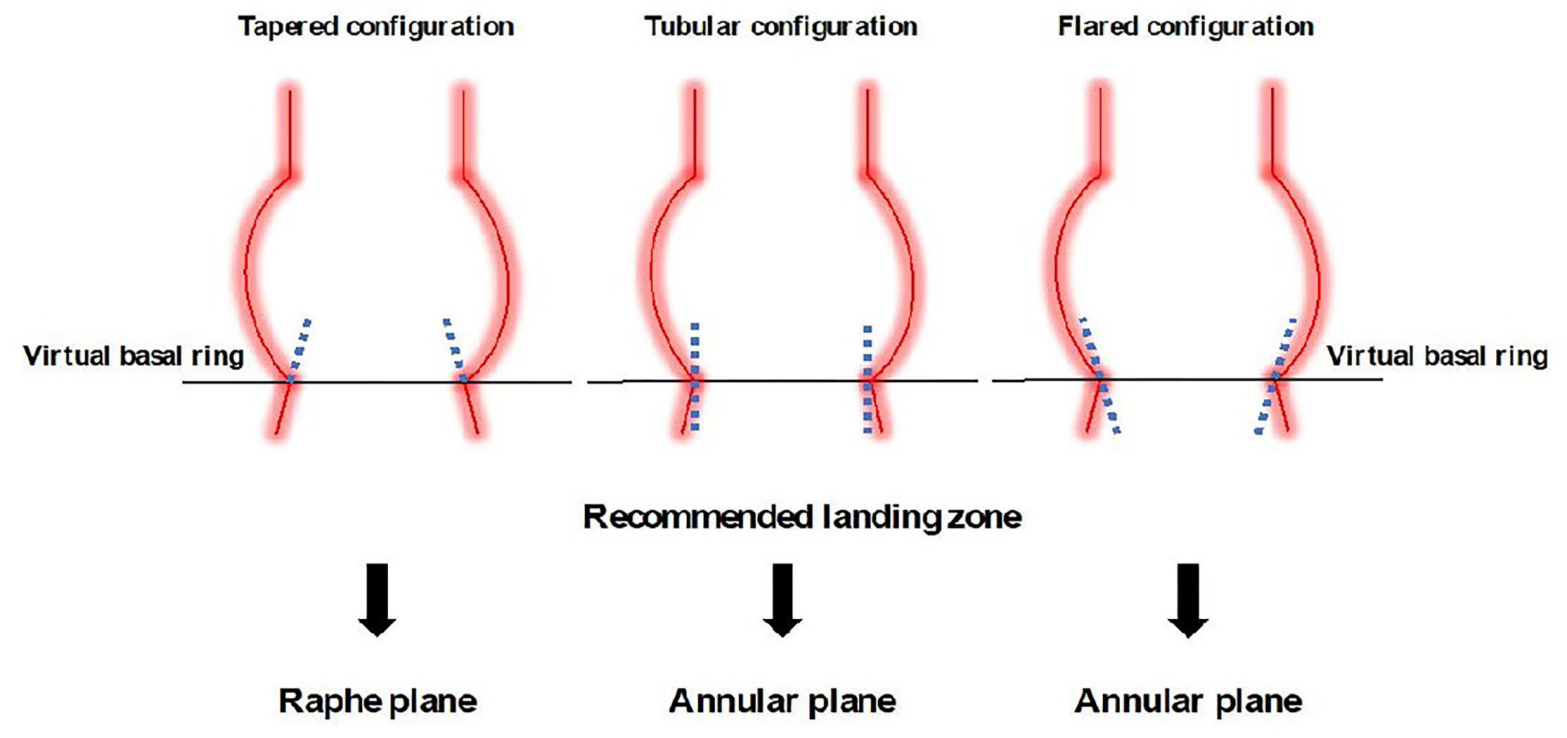
Recommended prosthesis sizing according to the aortic root morphology in BAVs.

The specific anatomical particularities of BAV highlight the importance of detailed aortic root analysis taking into account supra-annular structures (including calcification and raphe) in the prosthesis sizing process (Figure 3). A possible trend toward the prosthesis down-sizing according to standard measurements at the annulus level is to be considered, particularly in cases of tapered aortic root configuration. All these sizing algorithms need, however, further validation, namely, with special regards to the clinical outcomes according to different BAV morphologies (35).

### Evaluation of Coronary Obstruction Risk

Bicuspid aortic valve is associated with the higher coronary ostia take-off and larger sinuses of Valsalva (36). While these characteristics would rather prevent coronary obstruction, other specific characteristics of BAV have to be considered before TAVI. Excessive raphe calcification between the non-coronary cusp and the left or right coronary cusp may lead to the prosthesis displacement after deployment in the opposite direction obstructing either the left main or right coronary ostium. Furthermore, coronary ostia have been described closer to commissures leading to an increased risk of coronary obstruction, especially when leaflets are very asymmetrical or bulky (36). In case of a borderline situation despite the pre-procedural MSCT imaging analysis, balloon inflation with simultaneous aortography may identify aortic root at risk for coronary obstruction. Overall, the risk of coronary obstruction in BAV after TAVI of well-selected patients remains, however, low and similar to tricuspid valves (37). In the case of BAV anatomies at high risk for coronary obstruction, a similar to tricuspid valves approach is recommended, going from simple coronary pre-procedural wiring to chimney technique or Bioprosthetic or native Aortic Scallop Intentional Laceration to prevent Iatrogenic Coronary Artery obstruction during TAVR.

## Aortic Valve Replacement In BAV Stenosis

### Surgery

Comparison between TAVI and surgery for BAV relies on propensity-matched studies as no randomized trial exists yet. BAV stenosis was considered as an exclusion criterion in existing randomized studies comparing TAVI with surgery. Using Medicare data, Mentias et al. compared 699 matched pairs of BAV patients undergoing TAVI and surgery (38). In-hospital mortality and stroke rate were similar between TAVI and surgery (2.2 vs. 2.3%, *p* = 0.90/2.9 vs. 2.7%, *p* = 0.90/2.7 vs. 2.9%, *p* = 0.90, respectively). Thirty-day mortality and 1-year mortality were similar between both groups. Patients undergoing TAVI presented, however, a higher rate of new permanent pacemaker implantation in comparison with surgery (12.2 vs. 7.6%, *p* = 0.009, respectively). Interestingly, clinical outcomes remained similar after excluding patients undergoing concomitant coronary artery bypass graft or aortic root replacement surgery (38). A similar propensity-score matched study was conducted by Elbadawi et al. analyzing patients undergoing TAVI and isolated surgery for BAV (*n* = 975 pairs). Data were retrospectively collected from the US National Inpatient Sample database. After matching, in-hospital mortality and stroke rate were similar between TAVI and surgery (3.1 vs. 3.1 and 2.6 vs. 2.1%, respectively). Here again, patients undergoing TAVI had a higher permanent pacemaker implantation rate. The results of these 2 propensity-matched score studies are encouraging in the light of similar outcomes than studies randomizing patients with tricuspid valves to TAVI or surgery. However, dedicated randomized trials including patients with BAV still need to be designed. As TAVI indication has been progressively extended to younger patients, an increasing number of TAVI will be performed in BAV stenosis. Strong evidence is still lacking since patients with BAV were largely excluded from pivotal randomized trials. Registries of TAVI in BAV have reported excellent outcomes, though result interpretation is limited by significant selection bias related to registries. Before considering TAVI instead of surgery for most BAV stenosis, direct comparison between surgery and TAVI is mandatory, particularly when considering the excellent result of surgery in BAV. In addition, long-term outcomes will be needed with respect to the younger age of patients with BAV but data over 10 years are challenging to collect. In the latest and very recent ESC valvular heart guidelines (2021), the role of TAVI in BAV stenosis remains a gap of evidence, though the consensus paper considers a BAV as an unfavorable anatomical characteristic for TAVI (24). Interestingly, the U.S. FDA approved Edwards Sapien valve and Medtronic Corevalve for patients with aortic stenosis at low surgical risk patients in August 2019. At the same time, the Corevalve Evolut TAVI system obtained the approval for the treatment of BAV deemed at intermediate or greater risk for surgery followed by CE Mark and Health Canada approval, respectively, in June 2020 and January 2021.

## Transcatheter Aortic Valve Implantation

### Outcomes

Data reporting performances of TAVI in patients with BAV rely mainly on comparative retrospective and small prospective studies. Currently, the BAV stenosis candidates for TAVI are highly selected. Moreover, their younger age and reduced risk profile may definitively bias the comparison with tricuspid valve patients. The challenges raised by the non-standardized BAV patient selection process for TAVI may impact the procedural and clinical outcomes in-between the studies. Table 1 summarizes main studies reporting outcomes of TAVI in BAV using current generation devices (9, 15, 22, 35, 37, 39–43).

**Table 1:** Summarizes major published studies including >100 patients treated for bicuspid aortic valve (BAV) severe stenosis with the current generation of transcatheter heart valves.

	***N***	**Prosthesis**	**Aortic rupture, %**	**Conversion to surgery, %**	**≥Moderate PVL, %**	**PPM, %**	**Stroke, %**	**All-Cause mortality, %**
Yoon et al. (9)	102	S3 89% Lotus 11%	1	1	0	16.7	2	3.9
Yoon et al. (37)	226	S3 70.8% Lotus 19% Evolut R 10.2%	NA	1.3	2.7	16.4	3.2	3.7
Tchetche et al. (22)	101	S3 65.3% Evolut R 19.2% Lotus 9.9% Accurate neo 5.8% Other 1.9%	NA	NA	0 (severe)	13	2 (disabling)	0
Kim et al. (35)	184	S3 58.2% Accurate neo 26% Evolut R 7.1% Portico 6.5% Lotus 2.2%	1.1	1.6	4.3	14.5	4.3	3.2
Makkar et al. (39)	2691	S3 100%	0.3	0.9	2.1	9.1	2.5	2.6
Halim et al. (40)	3705	S3 86.7% Evolut R 13.3%	NA	0.7	2.4	NA	2	1.6 (in-hospital)
Forrest et al. (41)	932	Evolut R/PRO 100%	NA	0.6	7.7	15.4	3.4	2.6
Mangieri et al. (42)	353	S3 68.6% Evolut R/PRO 31.4%	1.1	NA	4	16.1	1.6	4.3
Yoon et al. (15)	1034	S3 71.6% Evolut R/PRO 18.2% Lotus 4.5% Accurate neo 3.9% Protico 1.8%	1.7	0.9	3.2	12.2	2.7	2
Forrest et al. (43)	150	Evolut R/PRO 100%	0	0.7	0	15.1	4	0.7

*NA, not available; PPM, permanent pacemaker; PVL, paravalvular leak*.

The largest report comes from the STS/ACC TVT registry (40). BAV stenosis represented 3.2% of the 170,959 TAVI procedures performed between 2011 and 2018. Patients with BAV were younger (74 vs. 82 years old, *p* < 0.001, respectively) with a lower risk profile in comparison with those with tricuspid valves. Although the device success (using only current-generation devices) was slightly lower in BAV than tricuspid valves with a higher incidence of ≥ moderate aortic regurgitation, 1-year mortality and stroke risk were not affected. Indeed, patients with BAV had a lower 1-year adjusted mortality [hazard ratio (*HR*), 0.88 (95% CI, 0.78–0.99)] with similar adjusted stroke rate [*HR*, 1.14 (95% CI, 0.94–1.39)] in comparison with patients with a tricuspid valve (40). Caution should be paid when interpreting the results in light of a statistically significant difference in prosthesis type used in BAV and tricuspid valves. Indeed, the Sapien 3 (Edwards Lifesciences, CA, USA) prosthesis was more frequently used in BAV (73 vs. 69%, *p* < 0.001, respectively), but remained the most used prosthesis in both groups (40).

A second analysis from the STS/ACC TVT registry analyzed data from all patients treated with the third-generation Sapien 3 prosthesis (Edwards Lifesciences) between 2015 and 2018. Similar 30-day [2.6 vs. 2.5% (95% CI, 0.74–1.47), respectively], and 1-year mortality [10.5 vs. 12.0% (95% CI, 0.73–1.10), respectively], were reported among 2,691 matched pairs of BAV and tricuspid valves (39). Stroke rate was, however, higher [2.5 vs. 1.6% (95% CI, 1.06–2.33)] and patients with BAV required more frequent open heart surgery conversion in comparison with tricuspid valves [0.9 vs. 0.4%, respectively, absolute risk difference 0.5% (95% CI, 0–0.9%)]. No difference in ≥ moderate aortic regurgitation was, however, reported at 30 days between both groups (39). More recent results of this registry were presented at the EuroPCR congress 2021 reporting outcomes of the same 3,168 propensity match pairs. Authors confirmed similar adjusted 1-year mortality (12 vs. 10.5%, *p* = 0.31) between BAV and tricuspid valves. Even though the stroke rate was higher at 30 days in the BAV group (2.4 vs. 1.6%, *p* = 0.02, respectively for BAV and tricuspid valves), the difference was no longer true when considering adjusted results. One-year stroke rate was similar among matched patients (3.4 vs. 3.1%, *p* = 0.16, respectively) (44).

Similarly, Forrest et al. analyzed data from all patients treated with the Evolut R or PRO valves (Medtronic) included in the STS/ACC TVT registry between 2015 and 2018. One-year all-cause mortality and stroke rate were similar between 1,858 matched pairs of BAV and tricuspid valves (10.4 vs. 12.4%, *p* = 0.63 and 3.9 vs. 4.4%, *p* = 0.93, respectively) (41). Interestingly, patients with BAV had higher rate of ≥ moderate aortic regurgitation post-procedure (5.6 vs. 2.1%, *p* < 0.001) but this difference was no longer significant at 1-year follow-up (4.7 vs. 3.9%, *p* = 0.60) (41).

A recent large meta-analysis compared outcomes between BAV and tricuspid valve among 17 studies and 181,433 patients undergoing TAVI, including 6,669 patients with BAV (0.27%). While the device success and 1-year mortality were similar between BAV and tricuspid valves in the matched population (97 vs. 94%, *p* = 0.55 and 91 vs. 91%, *p* = 0.22, respectively), patients had higher incidence of cerebral ischemic events (2.4 vs. 1.6%, *p* = 0.015) as well as moderate to severe aortic regurgitation (relative risk 1.42, *p* < 0.0001). Patients treated for BAV presented more frequent procedural complications with higher rate of annular rupture (*p* = 0.014) or conversion to surgery (*p* = 0.018) (45).

Finally, at the 2021 TVT structural heart summit, data from PARTNER 3 TAVI BAV registry were presented comparing 148 matched pairs of patients with BAV and tricuspid valves. No difference in terms of death, stroke, or rehospitalization were reported at 1 year between both anatomies (10.9 vs. 10.2%, *p* = 0.8, respectively, for BAV vs. tricuspid valves) (46).

Substantial iterative technical development of TAVI devices, in addition to the increasing experience and better preprocedural planning of operators, allowed for outcome improvement in BAV patients treated with current-generation devices. Indeed, in the STS/ACC TVT registry, the use of current-generation devices translated into device success increase and aortic regurgitation decline (40). A similar increase in device success and decrease in the paravalvular leak was already described in an earlier but smaller bicuspid TAVI international registry comparing outcomes of early- vs. new-generation devices (47). In a propensity score-matched study (*n* = 546 pairs) by Yoon et al. comparing TAVI in BAV vs. tricuspid valves, device success as well as mortality up to 2 years (17.2 vs. 19.4%, *p* = 0.28, respectively), was similar in patients receiving current generation devices (37).

Whereas, most of TAVI procedural complications and clinical outcomes in tricuspid aortic valve stenosis have significantly improved over time to reach non-inferiority if not superiority in comparison with surgery, high-grade conduction disorders remain a major issue post-TAVI. Several predictors of new permanent pacemaker implantation in tricuspid valves have been identified. Patient (such as baseline conduction disorders and aortic annulus anatomical characteristics) and procedural (such as, prosthesis oversizing, type, and implantation depth) characteristics are associated with an increased risk of high-grade conduction disorders (48, 49). The impact of valve morphology (BAV vs. tricuspid valves) on the new permanent pacemaker implantation rate is still controversial with conflicting results. Shorter membranous septum or asymmetrical radial forces of the prosthesis compressing the conduction system in BAV have been suggested as risk factors for conduction disorders (50). In the large STS/ACC TVT registry, permanent pacemaker implantation rate was slightly but significantly higher among the BAV matched to tricuspid valve patients (7.3 vs. 5.9%, *p* = 0.05, respectively), treated by the third generation Sapien 3 (Edwards Lifesciences) prosthesis (39). The difference became higher (9.1 vs. 7.5%, *p* = 0.03) in the recent up-to-date data presented at the 2021 EuroPCR congress (44). These results are in opposition to a recent meta-analysis including 19 studies (4,040 BAV vs. 8,084 tricuspid valves) where authors reported similar new permanent pacemaker implantation rates between both groups [risk ratio 1.06 (95% CI, 0.93–1.20)] (51). Device type (self-expandable vs. balloon-expandable) seems not to influence the pacemaker implantation rate among new-generation devices. Indeed, in the BEAT (balloon vs. self-expandable valve for the treatment of bicuspid aortic valve stenosis) international registry, BAV treated with self-expandable Evolut R/PRO (*n* = 111), or balloon-expandable Sapien 3 (*n* = 242) prosthesis were compared. The rate of permanent pacemaker was similar in both groups (16.0 vs. 16.1%, *p* = 0.98, respectively, for self- vs. balloon-expandable devices) (42). Interestingly, results remained similar after propensity-score matching. Higher rates of permanent pacemaker implantation were reported by Jilaihawi but, here again, with similar rates between self- and balloon-expandable devices (26.9 vs. 25.5%, *p* = 0.83, respectively) (14).

### Technical Considerations for TAVI in BAV

Specific technical considerations related to the different valve morphology and physiopathology in BAV are considered when considering TAVI. BAV opening orifice eccentricity increases the difficulty of retrograde valve crossing in case of severe stenosis. Fine analysis of MSCT pre-procedural imaging may help to identify the fused cusps and predict the location of wire crossing. A step-by-step approach has been suggested by Frangieh and Kasel starting from the non-fused cusp and rotating the catheter clockwise or counter-clockwise in case of left-right or non-right types, respectively (52). In case of no raphe type, no specific rule exists. When retrograde valve crossing remains impossible, transseptal puncture with anterograde aortic valve crossing can be performed.

Asymmetrical and increased burden of calcium deposition, and non-circular shape of BAV increase the risk of device malpositioning during the prosthesis deployment as well as the risk of annular rupture. Non-circular or valve underexpansion has been documented by imaging studies in BAV treated with both self- and balloon-expandable devices (53, 54). Use of the 2 orthogonal views after prosthesis implantation helps to identify the stent frame underexpansion that may be missed with a single fluoroscopic projection. The impact of prosthesis eccentricity on long-term valve function remains unestablished yet with no difference in hemodynamic parameters at short-term (17). In the BIVOLUT-X registry, systematic pre-dilatation (87% of the cases) and post-dilatation according to the angiography appearance of the prosthesis (55%) in BAV have shown favorable ellipticity index (1.2) with encouraging hemodynamic parameters of the self-expandable prosthesis at 30 days (mean gradient of 7.3 mmHg and ≥moderate paravalvular leak in 2% of the patients) (55). However, these anatomical challenges are to be better targeted in light of the higher rate of second valve implanted in BAV vs. tricuspid valves in the large STS/ACC TVT registry (40). The use of a recapturable device may here be a special interest in case of predicted challenging prosthesis deployment. Pre- and post-dilatation may help in optimizing the prosthesis landing zone at the price of an increased risk of annular rupture and stroke. In different analyses of the STS/ACC registry, the rate of conversion to open surgery was higher in BAV vs. tricuspid valves when a balloon-expandable device was used (0.9 vs. 0.4%, *p* = 0.03, respectively), whereas no significant difference was reported with self-expandable devices (0.6 vs. 0.2%, *p* = 0.29, respectively) (39, 41). When comparing self- to balloon-expandable devices in BAV, the BEAT registry reported higher rate of pre- and post-dilatation with self-expandable prosthesis (pre-dilatation: 57.3 vs. 37.9%; post-dilatation: 42.7 vs. 14.3%; *p* < 0.001 for both) (42). Balloon post-dilatation should be limited to cases with significant prosthesis dysfunction, including more than the mild paravalvular leak or mean gradient >15 mmHg. Indication for post-dilatation of non-circular valve geometry without a hemodynamic impact needs further investigations with long-term data on valve performances and leaflet thrombosis.

A horizontal aorta is frequently associated with BAV and may interfere with both retrograde valve crossing and prosthesis deployment (27). Although different techniques have been described to facilitate the valve crossing or delivery system orientation (56, 57), alternative accesses (transcarotid or axillary as the first alternative choices) can be decided at the time of pre-procedural planning (58).

Coronary re-access following TAVI in BAV is of particular interest according to the younger age of patients developing severe aortic stenosis in BAV in comparison with the tricuspid valve. However, similarly to tricuspid valves, no clear recommendation on commissural alignment during the prosthesis deployment exists yet. Eccentric coronary ostia in the leaflet as well as anomalous coronary origin may significantly complicate commissural alignment and thus coronary re-access in the future.

## Future Perspectives And Conclusions

**Figure 4:**
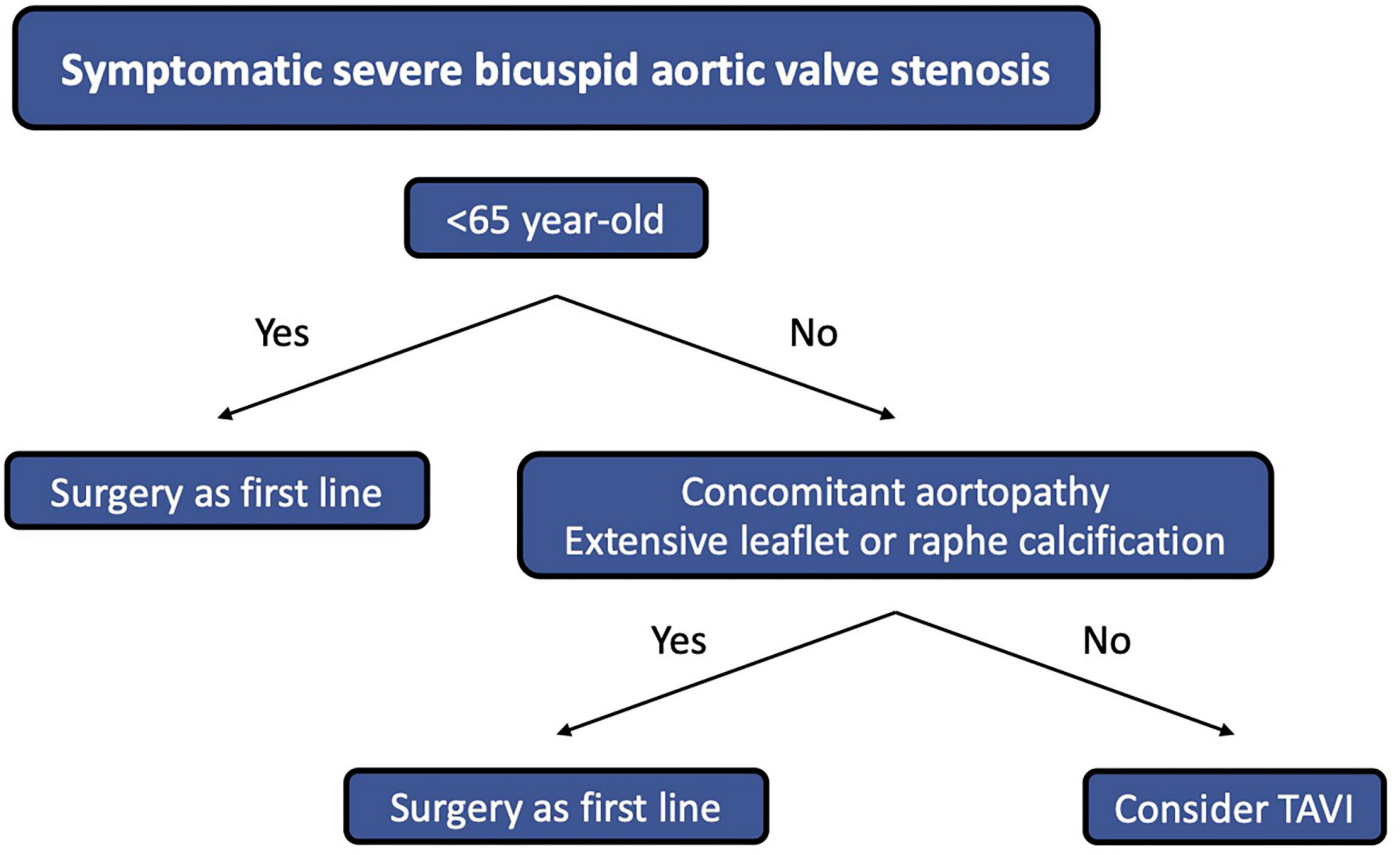
Treatment algorithm of patients with symptomatic severe BAV stenosis.

Data coming from specific designed randomized studies are needed to confirm the results of registries. To date, the NOTION-2 trial (NCT02825134) is randomizing low-risk patients with severe aortic stenosis to surgery or TAVI, such as BAV. A Chinese randomized non-inferiority trial (NCT03163329) comparing long-term results of TAVI and surgery in BAV is ongoing and results are expected by the middle of 2024. Long-term data assessing prosthesis hemodynamic performances over time are still lacking. Incomplete stent expansion or prosthesis distortion may influence the prosthesis durability and follow-up studies focusing on the structural valve failure and valve thrombosis become primordial with respect to the low-risk population of patients with BAV. Figure 4 suggests a treatment algorithm of patients with symptomatic severe BAV stenosis.

Bicuspid aortic valve is frequently associated with ascending aortopathy, such as aortic root and proximal ascending aorta dilatation. Currently, TAVI addresses only BAV stenosis and surgery remains the only option to treat the associated ascending aortopathy. The recent ESC guidelines recommend aortic root/proximal ascending aorta replacement in case of a diameter ≥ 45 mm when surgery is planned for BAV severe stenosis (24). If those patients are deemed inoperable, TAVI may be considered for the aortic stenosis, taking into account the higher risk of aortic dissection in this setting (59, 60). Whereas, it is well-known that aortic root dilatation progresses with time in BAV, the rate of progression after TAVI remains unknown. Protheses treating aortic valve and ascending aortopathy simultaneously (Endo-Bentall) are under development with encouraging first-in-man cases, however, reserved for compassionate use yet (61).

In conclusion, BAV stenosis has distinct anatomical characteristics in comparison with tricuspid valves leading to specific aortic root distortion. Several sub-types classifications have been developed over time to better address the therapeutic options. When TAVI is considered for BAV, pre-procedural MSCT imaging is essential to assess the number of cusps, presence of a raphe, and location of calcifications. Aortic root, such as supra-annular structures, should be integrated in the device selection and sizing process as prosthesis interaction with the aortic root can occur from the level of the left ventricular outflow tract to above the sinotubular junction. Favorable clinical and safety outcomes have been reported from large international registries with similar outcomes in comparison with tricuspid valves. However, data from randomized trials are needed.

## Author Contributions

NP contributed to the design of the review and writing of the manuscript. RI, ND, AB, LL, PD, and TM revised the manuscript. WB contributed to the design of the review and reviewed critically the manuscript. All authors contributed to the article and approved the submitted version.

## Funding

NP has received research support from the Swiss National Science Foundation (P400PM_194483) and the Geneva University Hospitals.

## Conflict of Interest

The authors declare that the research was conducted in the absence of any commercial or financial relationships that could be construed as a potential conflict of interest.

## Publisher's Note

All claims expressed in this article are solely those of the authors and do not necessarily represent those of their affiliated organizations, or those of the publisher, the editors and the reviewers. Any product that may be evaluated in this article, or claim that may be made by its manufacturer, is not guaranteed or endorsed by the publisher.
